# Supplementary figures and images for: Early Bolting, Yield, and Quality of Angelica sinensis (Oliv.) Diels Responses to Intercropping Patterns
Source: Plants (Basel). 2022 Nov 1;11(21):2950. doi: 10.3390/plants11212950 (PMC9657888; doi:10.3390/plants11212950)

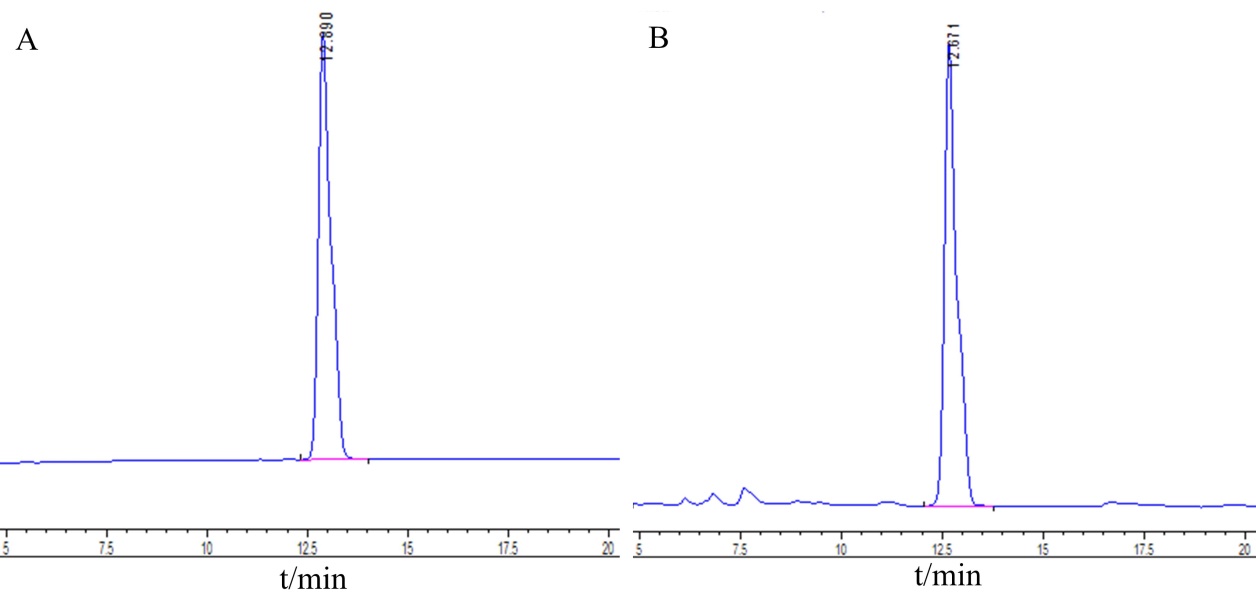

Supplement: Supplementary file 1 [file plants-11-02950-s001.zip › Figure S1.jpg]

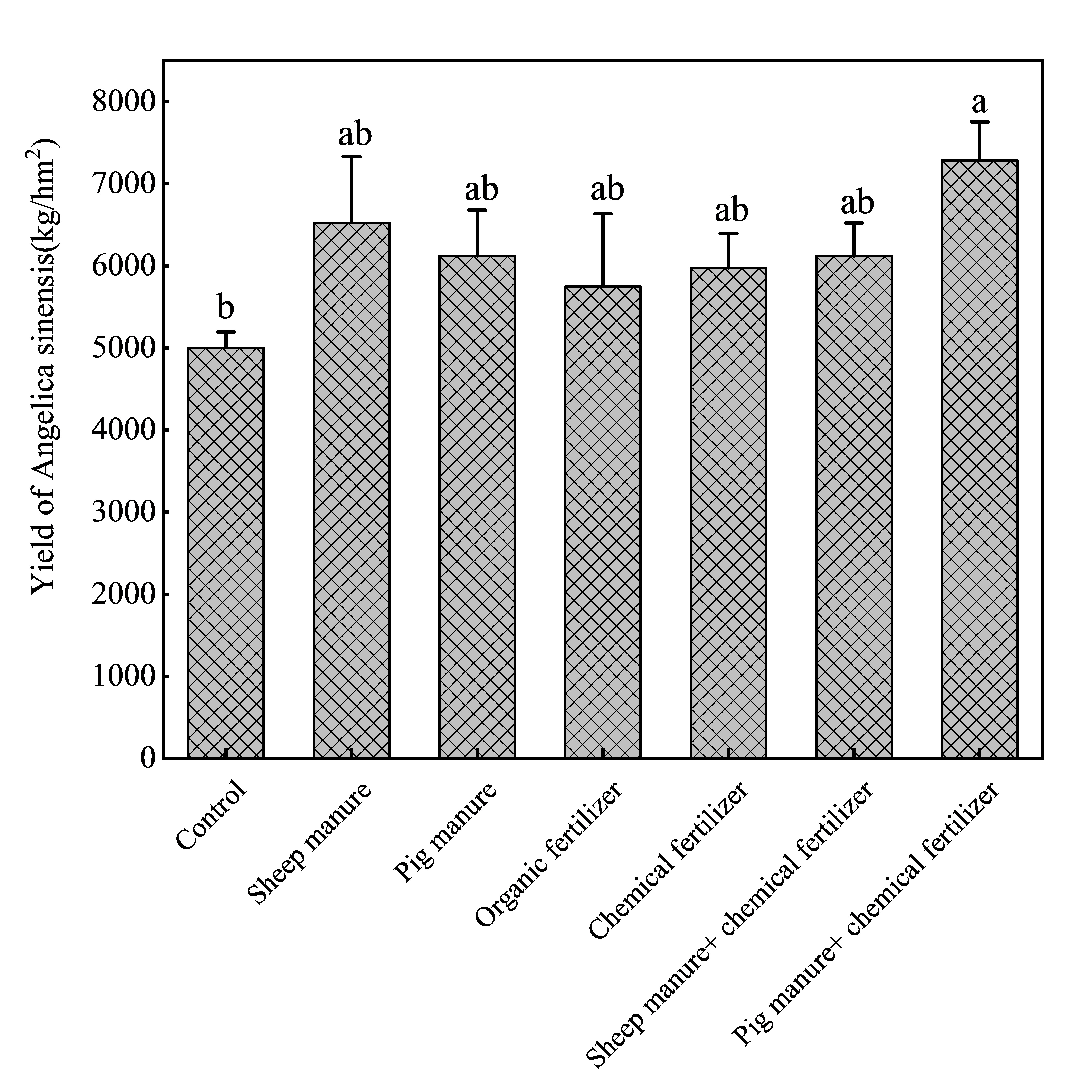

Supplement: Supplementary file 1 [file plants-11-02950-s001.zip › Figure S2.jpg]
